# The relationship between family adaptability and cohesion and adolescent depression: the chain mediation effect of perceived social support and coping styles

**DOI:** 10.3389/fpsyt.2026.1775775

**Published:** 2026-02-20

**Authors:** Jiuju Li, Xinyue Chen, Zixuan Zhang, Jiale Zhao, Libo Ai, Ying Zhang, Li Li, Ruixin Wang

**Affiliations:** 1 Beijing Huilongguan Hospital, Capital Medical University, Beijing, China; 2 School of Psychology and Mental Health, North China University of Science and Technology, Tangshan, China; 3 Nursing Department, Shanghai Mental Health Center, Shanghai, China

**Keywords:** adolescent, coping styles, depression, family adaptability and cohesion, perceived social support

## Abstract

**Purpose:**

The research aims to investigate the relationship between family adaptability and cohesion and adolescent depression by constructing a chain mediation model to examine the mediating role of perceived social support and coping styles.

**Participants and methods:**

This study surveyed 1,931 middle school students using the Family Adaptability and Cohesion Evaluation Scale (FACES II-CV), the Perceived Social Support Scale (PSSS), the Simple Coping Style Questionnaire (SCSQ), and the Patient Health Questionnaire-9 (PHQ-9). Data were analyzed with SPSS 26.0, including tests for common method bias, descriptive statistics, and correlation analysis. The chain mediation model was tested using the PROCESS macro program.

**Results:**

Family adaptability and cohesion, perceived social support, and positive coping styles all showed significant negative correlations with adolescent depression, while negative coping styles exhibited a positive association with depression. Furthermore, family adaptability and cohesion not only have a direct impact on adolescent depression, but also exert indirect effects through the separate mediating roles of perceived social support, positive and negative coping, as well as through the chain mediation of “perceived social support → positive/negative coping styles.” Among these pathways, the two chain mediation paths (Family adaptability and cohesion → Perceived social support (PSS) → Positive coping styles → Depression; Family adaptability and cohesion → Perceived social support (PSS) → Negative coping styles → Depression) accounted for 21.43% and 7.14% of the total effect, respectively.

**Conclusion:**

This study clarifies the relationship between family adaptability and cohesion, PSS, coping styles, and adolescent depression, emphasizing the indirect influence of family adaptability and cohesion on adolescent depression through PSS, positive coping, and negative coping styles. To a certain extent, it offers evidence and theoretical guidance for mitigating and treating adolescent depression.

## Introduction

Depression is one of the key criteria for assessing an individual’s mental health status, specifically manifested as persistent negative emotional experiences in daily life (1). Currently, depression has become one of the most prevalent mental illnesses worldwide, with the adolescent demographic warranting particular attention. According to statistics, the global prevalence of depression among adolescents stands at 8%. Meanwhile, the current detection rate of depression among adolescents in China reaches 28.4%, which is notably higher than the international average (2). These figures highlight the severity of adolescent depression in China, underscoring the urgency of addressing this issue. The initial teenage years is a pivotal phase for the emergence of depressive symptoms (3). Adolescents are more prone to various emotional problems, including depression, due to their immature physical and mental development and the many social development tasks they face (4). Depression places a heavy burden on individuals, society, and the economy during this critical period of life transition. It not only triggers issues such as substance use, sleep disturbances, and eating problems among adolescents, but also brings great psychological distress, severely undermining their quality of life, and even leads to self-harm and suicidal behaviors, increasing the risk of lifelong physical and mental health damage in adolescents (5). Therefore, it is crucial to conduct in-depth research on the factors influencing adolescent depression symptoms and their mechanisms of action, identify protective factors that can reduce the occurrence of depression symptoms at an early stage, and take timely intervention measures.

### The impact of family adaptability and cohesion on symptoms of depression

The concepts of family adaptability and cohesion were first proposed by Olson and his research team in 1979, based on Bowen’s family systems theory. Family cohesion reflects the closeness of emotional connections between members, while adaptability concerns the capacity of a family system to modify itself in reaction to challenges encountered across various phases of family growth and diverse familial contexts (6). These two variables can be used to assess the health and stability of a family, measure the strength of relationships between family members, and evaluate their ability to cope with challenges. The circumplex model theory of family functions proposes that balanced family cohesion is most conducive to mental health (7). For adolescents, insufficient family cohesion may increase the risk of emotional neglect, whereas excessive closeness may inhibit individual autonomy. Both factors are associated with higher levels of depression and ultimately contribute to depressive tendencies. Families with higher scores in adaptability and cohesion, on the one hand, provide adolescents with a sense of security, reducing feelings of loneliness and helplessness, thereby buffering depressive emotions. On the other hand, close family relationships are often correlated with more effective stress coping abilities, both of which collectively serve as protective factors against depression risk. Conversely, lower scores indicate poor family functioning, which may involve family conflicts, emotional detachment, or excessive control. These conditions are often linked to higher tendencies of self-blame and feelings of worthlessness in adolescents, thereby increasing vulnerability to depression. Empirical research also indicates that family conflict and intimacy are important predictors of adolescent mental health. Adolescents with higher family cohesion are less likely to develop depressive or other internalizing issues (8).

Therefore, we propose hypothesis H1: Family adaptability and cohesion negatively predict adolescent depression.

### The mediating effect of PSS

The social support perceived by an individual means the individuals’ satisfaction with the degree of support they receive from society and individuals’ expectations about the social support they are likely to receive (9). This expectation often begins in the family environment, because the family is an important place for individuals’ physical and mental development. This is particularly important for young people, whose family is their main source of material and emotional support. Family Systems Theory suggests that high-cohesion families can provide a stable support network, with family members internalizing this supportive experience and generalizing it to other social relationships (10). This theoretical perspective is supported by empirical research, and many studies have clearly shown that there is a significant positive correlation between family cohesion and PSS (11, 12). For adolescents in the critical period of psychological development, their perception of social support is profoundly influenced by the family environment. Studies have shown that adolescents from families with higher cohesion tend to exhibit greater PSS compared to their peers from less intimate family settings (13), thereby experiencing more positive and healthy emotions. In highly cohesive families, members can rely on available social support (14). Meanwhile, the main effect model of social support (15) further indicates that PSS is associated with lower levels of negative emotional states. Previous research has confirmed a significant negative correlation between PSS and depression among adolescents (16), suggesting that stronger perception of social support corresponds to greater experienced support and lower susceptibility to depressive emotions (17). Furthermore, a longitudinal study also found that among adolescents with lower family relationship quality, their internal cognition and emotional experiences are more negative, their perceived level of social support is lower, and they also exhibit a higher tendency toward depression (18).

Therefore, we propose hypothesis H2: PSS mediates the relationship between family adaptability and cohesion and adolescent depression.

### The mediating effect of coping styles

Coping styles refer to a series of strategies that individuals adopt in order to adapt to the needs of the new environment when facing pressure and external events (19). The interaction theory of coping styles emphasizes that stress coping is a dynamic, ongoing process of interaction between the individual and the environment, with strategies changing according to situational demands and the individual’s state (20). As the family environment serves as the most fundamental and enduring contextual factor in adolescent growth and development, adolescents’ coping styles are likely closely associated with their family surroundings. A study on adolescent mental health intervention indicates that the effectiveness of intervention measures in improving adolescents’ internalizing problems is partially achieved by promoting positive parenting behaviors and enhancing adolescents’ positive coping strategies (21). Specifically, higher levels of family adaptability and cohesion are associated with a greater tendency among adolescents to employ positive coping styles (22). Conversely, when families lack emotional connection, adolescents may feel isolated and unsupported, making them more prone to adopt negative coping strategies. Coping style is an important dimension for understanding adolescent depression. Cognitive theory of depression (23) posits that the coping strategies individuals adopt in response to stress are closely linked to their mental health status. Research has shown a significant positive correlation between negative coping styles and depression (24), with negative coping being associated with more negative emotional experiences, whereas positive coping is related to better psychological adjustment (25). For example, in stressful situations, adolescents who use positive coping strategies can more effectively mitigate the adverse emotions caused by negative events, thereby enhancing their subjective well-being (26). Thus, a family environment characterized by higher cohesion and adaptability may encourage adolescents to adopt more positive coping strategies, which aids in emotion regulation and is associated with lower levels of depression. In contrast, adolescents from families with lower cohesion are more inclined to use negative coping strategies, potentially increasing the occurrence of negative emotions and elevating the risk of depression.

Therefore, we propose hypothesis H3: coping styles mediate the relationship between family adaptability and cohesion and adolescent depression.

### The chain mediating effect of PSS and coping styles

PSS reflects their close connection with all aspects of society. Coping styles refer to the cognitive or behavioral strategies that individuals use to regulate emotions or solve problems when facing stressful events. The buffering model suggests that social support primarily serves as a “buffer” when individuals face high levels of stress, prompting them to adopt more adaptive coping styles to address stressful situations (15). This reduces the negative emotional impact of stress, such as anxiety, and improves mental health. Adolescents with a higher sense of support are more likely to choose positive coping styles, while those who feel they lack social support are more likely to choose negative coping styles. Research has shown that when adolescents perceived adequate social support, they were more likely to get practical help and emotional comfort from their social circles. This made them more likely to respond to stress and challenges in a proactive manner (27). In such cases, they typically experience fewer negative emotions. Conversely, adolescents with lower PSS often feel helpless and tend to resort to negative coping strategies (28), a pattern commonly associated with higher levels of depression. Therefore, according to the buffer model, the family as the primary source of support means that family cohesion is closely related to adolescents’ subjective perception of social support quality (29). High family cohesion is often accompanied by greater PSS, which in turn promotes the use of positive coping strategies, leading to better emotional regulation outcomes and lower levels of depression. In contrast, adolescents from low-cohesion families tend to perceive less social support, making them more likely to adopt negative coping strategies, which in turn is linked to a higher risk of depression.

Therefore, we propose hypothesis H4: PSS and coping styles play a chain mediating role in the influence of family adaptability and cohesion on adolescent depression.

### Theoretical framework

Based on Family Systems Theory, the Social Support Buffering Model, and the Cognitive Theory of Depression, this study constructed an integrated chain mediation model to systematically elucidate the sequential mechanisms through which the family environment influences adolescent depression. First, this research incorporates two core dimensions of family functioning—family cohesion and family adaptability—into the predictive model as key variables for adolescent depression. This integrated perspective enables a more comprehensive and systematic assessment of the impact of the family environment on adolescent mental health compared to previous studies. Second, whereas prior research on the relationship between family functioning and adolescent depression has predominantly focused on examining single mediation pathways (30), this study integrates two mediation pathways based on the Buffering Model, constructing a sequential mediation model of “family adaptability and cohesion → PSS → coping styles → adolescent depression.” This model reveals the continuous mechanism by which the family environment shapes individual behavioral strategies through social cognition, thereby affecting psychological well-being. This finding deepens our understanding of the internal mechanisms through which family functioning influences adolescent depression, integrating previously fragmented findings into a more systematic and dynamic theoretical framework, thereby offering new directions and insights for future research. Finally, in terms of research perspective, unlike previous studies that approached the causes of adolescent depression from a single perspective—whether individual, family, or social—this study organically integrates factors from the family, social, and individual levels, systematically examining their combined and interactive effects (31–33). This approach provides a more comprehensive understanding of the formation mechanisms of adolescent depression and offers a stronger theoretical foundation for subsequent intervention and practical applications.

Furthermore, while focusing on the core variables, this study also considered potentially relevant demographic variables. Existing research has indicated that demographic factors such as student gender, primary caregiver’s gender, and their educational level may be associated with family resources, parenting styles, and the level of attention given to mental health, thereby potentially influencing the psychological development environment and emotional state of adolescents (34, 35). Therefore, in order to more clearly reveal the intrinsic relationships among family adaptability and cohesion, PSS, coping styles, and depression, this study controlled demographic variables in the subsequent mediation effect analyses.

**Figure 1:**
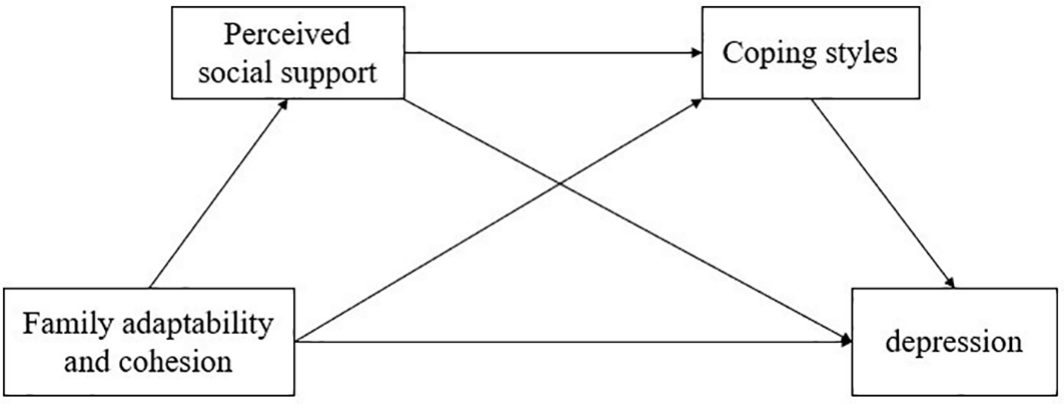
Theoretical model.

Overall, the purpose of this study is to explore the association between family adaptability and cohesion and depression. This study also examines the chain-mediated role of PSS and coping styles between family adaptability and cohesion and depression. The theoretical framework of this study is shown in **Figure 1**.

## Material and methods

### Participants and procedure

To estimate the population proportion and determine the required sample size, this study adopted the classical formula proposed by Cochran for calculating the minimum sample size (36):


$$n=\frac{{\text{Z}}^{2}*\text{p}(1-\text{p})}{{\text{e}}^{2}}$$

Where n denotes the required sample size; Z denotes the critical value of the standard normal distribution, typically 1.96 for a 95% confidence level; p denotes the expected population proportion, typically conservatively assumed to be 0.5; and e denotes the maximum allowable error range, which is usually set to ±5% in studies (36). The final calculation yields a minimum required sample size of 384.

A survey was conducted via convenience sampling on 2,026 adolescent students aged 10–19 from a secondary school. Among the collected questionnaires, 95 were determined to be invalid due to missing answers or patterned responding, and were eliminated. In the end, This study received 1,931 valid questionnaires, and the overall response rate reached 95.31%. Among these, there were 561 males (29.1%) and 1,370 females (70.9%), with an average age of 15.39 ± 2.03 years; Among the primary caregivers, there were 398 males (20.6%) and 1,533 females (79.4%); Among primary caregivers’ educational levels, 68 were illiterate (3.5%), 337 had a primary school education (17.5%); 606 had a junior high school education (31.4%), 289 had a high school or vocational school education (15.0%), 578 had a college education (29.9%), and 53 had a graduate degree or higher (2.7%). **Table 1** is a demographic survey data table.

**Table 1:** General demographic survey sata form (*n* = 1931).

Variable name	Categories	Frequency	Percentage
Gender	Male	561	29.1%
	Female	1370	70.9%
Gender of primary caregiver	Male	398	20.6%
	Female	1533	79.4%
Educational attainment of primary caregivers	Illiterate	68	3.5%
	Primary school	337	17.5%
	Junior high school	606	31.4%
	High school or vocational school	289	15.0%
	College	578	29.9%
	Graduate degree or higher	53	2.7%

This study has obtained ethical approval from the Ethics Committee of Beijing Huilongguan Hospital (Approval No. 2023-79-Ke). Written informed consent was obtained from all participants and their legal guardians. The study adhered to the principle of voluntary participation, and participants were free to withdraw from the survey at any time.

## Measures

### Family adaptability and cohesion

This study used the Chinese version of the Family Adaptability and Cohesion Evaluation Scale (FACES II-CV) compiled by Olson and revised by Fei Lipeng et al (37). The original scale consists of two parallel subscales: one measuring individuals’ perceptions of their actual family circumstances and the other assessing their ideal family circumstances. Both subscales contain 30 identical items. For the purposes of this study, considering response convenience and the practical significance of the results, only the subscale pertaining to perceptions of the actual family circumstances was utilized. This scale comprises 30 items and measures two dimensions: family cohesion and family adaptability. The intimacy subscale contains 16 specific items, and the adaptability subscale contains 14 specific items. The questionnaire uses a five-level scoring method. The higher the score, the higher the level of family cohesion and adaptability. The Cronbach’s alpha coefficient for this scale is 0.945, while the coefficients for the family cohesion and adaptability dimensions were found to be 0.880 and 0.907, respectively.

### PSS

This study employed the Perceived Social Support Scale (PSSS) developed by Zimet et al. (38), a measure designed to assess an individual’s self-perceived and subjectively interpreted social support. The scale evaluates the extent of support perceived by individuals from various sources of social support—such as family, friends, and others—while the total score reflects the overall level of social support experienced by the individual. The scale contains 12 specific items and is scored using a 7-level Likert scale (1 represents strong disagreement and 7 represents strong agreement). The higher the total score of the scale, the higher the level of social support perceived by the individual. The overall Cronbach’s Alpha coefficient of this scale reaches 0.933. The Cronbach’s α coefficients for the family support, friend support, and other support subscales were 0.905, 0.928, and 0.873, respectively.

### Coping styles

The study utilized the Simple Coping Style Questionnaire (SCSQ) developed by Xie Yaning (39). This scale contains 20 specific items, adopts a four-point scoring system, and is composed of two dimensions: positive coping and negative coping. Among them, the first 12 items belong to the Positive Coping dimension (e.g., “Try to see the good side of things”), while the last 8 items constitute the Negative Coping dimension (e.g., “Accept reality because there is no other way”). The Cronbach alpha coefficient of the entire scale reached 0.875, of which the Cronbach’s alpha coefficient of the positive coping dimension was 0.892, and the Cronbach’s alpha coefficient of the negative coping dimension was 0.727.

### Depression

This study used the Patient Health Questionnaire-9 (PHQ-9) (40). This scale contains a total of nine items, each item using a four-level scoring method (from 0 to 3). The total score of the entire scale ranges from 0 to 27 points. The total score is used to assess the severity of depressive symptoms, with higher scores indicating more pronounced depressive symptoms. The Cronbach’s alpha coefficient for the total scale was 0.933.

### Statistical analysis

This study used SPSS26.0 statistical software to conduct common method deviation test, independent sample t-test, one-way analysis of variance, and correlation analysis on the collected data. Hayes’ SPSS macro program PROCESS3.5 model 6 was used to test chained mediating effects. Meanwhile, in the data analysis, categorical covariates were dummy-coded, with sex and primary caregiver sex assigned as male=0 and female=1. These covariates were included as control variables in the chain mediation analysis to control for their effects on depression.

## Results

### Common method bias

This study used Harman’s one-factor test to assess common method deviations, and all scale items were subjected to an unrotated exploratory factor analysis in this investigation. The results showed that there were 9 factors with eigenvalues greater than 1, and the maximum variance explained by the first factor was 29.72%, which was below the 40% threshold. Therefore, the common method deviations in this study was within an acceptable range.

### Correlation analysis

Pearson correlation analysis revealed the following results (shown in **Table 2**): family adaptability and cohesion were significantly positively correlated with PSS, positive coping, and negative coping (*r* = 0.564, 0.478, 0.197, *p* < 0.01), while family adaptability and cohesion showed a significant negative correlated with depression (*r* = -0.301, *p* < 0.01). PSS was positively correlated with positive and negative coping styles (*r* = 0.533, 0.246, *p* < 0.01) and negatively correlated with depression (*r* = -0.335, *p* < 0.01). Positive coping styles were negatively correlated with depression (*r* = -0.389, *p* < 0.01), while negative coping styles were positively correlated with depression (*r* = 0.080, *p* < 0.01). Therefore, Hypothesis 1 is supported.

**Table 2:** Descriptive statistics and correlation analysis of variables (*n* = 1931).

Variable name	*M ± SD*	FAC	PSS	PCS	NCS	Depression
FAC PSS PCS NCS Depression	81.40 ± 24.87 51.39 ± 15.79 16.39 ± 8.19 12.37 ± 5.10 16.00 ± 7.89	1 0.564** 0.478** 0.197** -0.301**	1 0.533** 0.246** -0.335**	1 0.427** -0.389**	1 0.080**	1

**p < 0.01. The same applies below.

FAC, family adaptability and cohesion; PSS, perceived social support; PCS, positive coping styles; NCS, negative coping styles. The same applies below.

### Differences in family adaptability and cohesion, PSS, coping styles, and adolescent depression scores across different demographic variables.

Independent samples t-tests were conducted to examine differences in family adaptability and cohesion, PSS, coping styles, and adolescent depression by student gender and primary caregiver gender. The results revealed significant student gender differences in family adaptability and cohesion, PSS, positive coping style, and adolescent depression (*t* = 4.50, 2.92, 4.47, -9.70, all *p* < 0.01). Additionally, significant differences were found in family adaptability and cohesion, PSS, and adolescent depression across the gender of the primary caregiver (*t* = 3.08, 2.25, -3.38, all *p* < 0.05). These findings indicated that male students and adolescents with a male primary caregiver scored significantly higher on family adaptability and cohesion as well as PSS; meanwhile, male students also obtained significantly higher scores on positive coping styles than female students. In contrast, female students and adolescents with a female primary caregiver exhibited higher levels of depressive symptoms.

A one-way analysis of variance was performed to test for differences in family adaptability and cohesion, PSS, coping styles, and adolescent depression based on the educational level of the primary caregiver. The results showed no significant differences in negative coping styles and adolescent depression across the educational level of the primary caregiver (*F* = 1.71, 1.00, all *p* > 0.05), whereas significant differences were identified in family adaptability and cohesion, PSS, and positive coping styles (*F* = 14.49, 7.94, 5.67, all *p* < 0.01). *Post-hoc* tests further demonstrated that the higher the educational level of the primary caregiver, the higher the scores of adolescents on family adaptability and cohesion, PSS, and positive coping styles.

### Analyzing the chain mediation effect of PSS and coping styles

**Figure 2:**
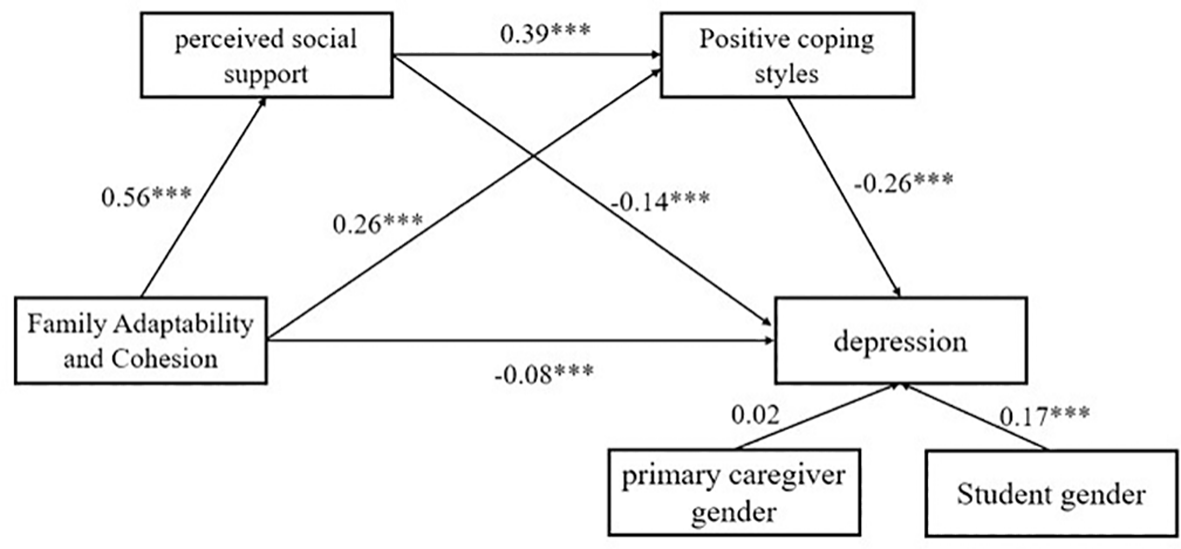
The Chain mediation model of PSS and positive coping style (***p < 0.001).

Independent samples t-tests and one-way analysis of variance revealed significant differences in adolescent depression based on both student gender and primary caregiver gender (all *p* < 0.05). Consequently, in the subsequent chain mediation analysis, these demographic variables were included as covariates, after being dummy-coded. The results are presented as follows:

After controlling for student gender and primary caregiver gender, we found that Family adaptability and cohesion significantly and positively predicted PSS (*β* = 0.56, *SE* = 0.02, *t* = 29.73, *p* < 0.001) and positive coping styles (*β* = 0.26, *SE* = 0.02, *t* = 11.30, *p* < 0.001), and negatively predicted adolescent depression (*β* = -0.08, *SE* = 0.03, *t* = -3.16, *p* < 0.001). PSS significantly and positively predicted positive coping styles (*β* = 0.39, *SE* = 0.02, *t* = 17.08, *p* < 0.001) and negatively predicted adolescent depression (*β* = -0.14, *SE* = 0.03, *t* = -5.33, *p* < 0.001). Positive coping styles also significantly negatively predicted adolescent depression (*β* = -0.26, *SE* = 0.02, *t* = -10.42, *p* < 0.001). The mediation effect results are presented in **Tables 3**, **4**, indicating that PSS and positive coping styles each play a partial mediating role in the relationship between family adaptability and cohesion and adolescent depression, accounting for 28.57% and 25% of the total effect, respectively. Additionally, the chain mediating effect of PSS and positive coping style was also significant, contributing to 21.43% of the total effect, as detailed in **Figure 2**.

**Table 3:** The chain mediation analysis of the pathway between PSS and positive coping styles.

Predictor variable	Depression		PSS		PCS		Depression	
	*β*	*t*	*β*	*t*	*β*	*t*	*β*	*t*
Student gender	0.18	8.37***	-0.01	-0.35	-0.05	-2.65***	0.17	8.07***
Primary caregiver gender	0.02	0.88	-0.01	-0.53	0.01	0.29	0.02	0.88
FAC	-0.28	-13.12***	0.56	29.73***	0.26	11.30***	-0.08	-3.16***
PSS					0.39	17.08***	-0.14	-5.33***
PCS							-0.26	-10.42***
*R^2^*	0.13		0.32		0.33		0.21	
*F*	92.34		300.06		239.56		102.02	

***p < 0.001.

**Table 4:** Positive coping styles mediation effect, direct effect, and total effect decomposition diagram.

Path	Effect	*Boot SE*	*Boot CI*	Effect proportion
FAC → depression	-0.08	0.03	[-0.13,-0.03]	28.57%
FAC → PSS → depression	-0.08	0.02	[-0.11,-0.05]	28.57%
FAC → PCS → depression	-0.07	0.02	[-0.09,-0.05]	25%
FAC → PSS → PCS → depression	-0.06	0.01	[-0.07,-0.04]	21.43%
Total indirect effect	-0.20	0.02	[-0.24,-0.17]	71.43%
Total effect	-0.28	0.02	[-0.32,-0.24]	

**Figure 3:**
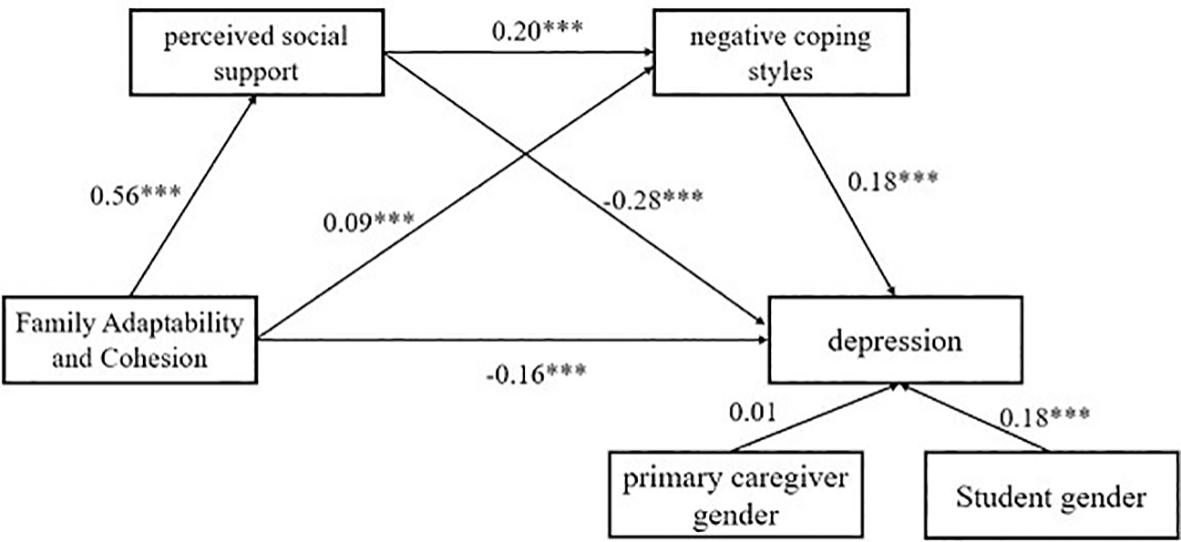
The chain mediation model of PSS and negative coping style (***p < 0.001).

Family adaptability and cohesion have a significant and positive predictive effect on the PSS (*β* = 0.56, *SE* = 0.02, *t* = 29.73, *p* < 0.001) and negative coping styles (*β* = 0.09, *SE* = 0.03, *t* = 3.34, *p* < 0.001), and it negatively predicted adolescent depression (*β* = -0.16, *SE* = 0.02, *t* = -6.51, *p* < 0.001). PSS significantly and positively predicted negative coping styles (*β* = 0.20, *SE* = 0.03, *t* = 7.44, *p* < 0.001) and negatively predicted adolescent depression (*β* = -0.28, *SE* = 0.02, *t* = -10.59, *p* < 0.001). Negative coping significantly positively predicted adolescent depression (*β* = 0.18, *SE* = 0.02, *t* = 3.34, *p* < 0.001). The mediation effects are presented in **Tables 5**, **6**, indicating that PSS and negative coping style each play a partial mediating role in the relationship between family adaptability and cohesion and adolescent depression, accounting for 53.57% and 7.14% of the total effect, respectively. Additionally, the chain mediation effect through PSS and negative coping style was also significant, contributing to 7.14% of the total effect, as illustrated in **Figure 3**. Therefore, hypotheses 2, 3, and 4 are supported.

**Table 5:** The chain mediation analysis of the pathway between PSS and negative coping styles.

Predictor variable	Depression		PSS		NCS		Depression	
	*β*	*t*	*β*	*t*	*β*	*t*	*β*	*t*
Student gender	0.18	8.37***	-0.01	-0.35	0.02	1.10	0.18	8.42***
Primary caregiver gender	0.02	0.88	-0.01	-0.53	0.03	1.18	0.01	0.57
FAC	-0.28	-13.12***	0.56	29.73***	0.09	3.34***	-0.16	-6.51***
PSS					0.20	7.44***	-0.28	-10.95***
NCS							0.18	8.42***
*R^2^*	0.13		0.32		0.07		0.19	
*F*	92.34		300.06		34.55		93.02	

***p < 0.001.

**Table 6:** Negative coping styles mediation effect, direct effect, and total effect decomposition diagram.

Path	Effect	*Boot SE*	*Boot CI*	Effect proportion
FAC → depression	-0.16	0.02	[-0.21,-0.11]	57.14%
FAC → PSS → depression	-0.15	0.02	[-0.19,-0.13]	53.57%
FAC → NCS → depression	0.02	0.01	[0.01,0.03]	7.14%
FAC → PSS → NCS → depression	0.02	0	[0.01,0.03]	7.14%
Total indirect effect	-0.12	0.02	[-0.15,-0.09]	42.86%
Total effect	-0.28	0.02	[-0.32,-0.24]	

## Discussion

This study examined the impact of family adaptability and cohesion on depression, with a particular focus on the chain-mediated role of PSS and coping styles. The aim was to identify factors influencing adolescent depression in order to develop effective intervention measures, reduce the incidence of depression among adolescents, and protect their mental and physical health. The results of this study clearly show that there is a negative correlation between family adaptability and cohesion and adolescent depression. In other words, the higher the level of family adaptability and cohesion, the lower the level of depression that adolescents will show. PSS and coping styles play a chain-mediated role in the relationship between family adaptability and cohesion and adolescent depression. Adolescents with higher family adaptability and cohesion have higher levels of PSS and are more likely to adopt positive coping styles, thereby reducing depression levels. Meanwhile, adolescents with stronger family cohesion and greater social support may also be more prone to adopting negative coping strategies, which ultimately heightens their risk of depression.

### The impact of family adaptability and cohesion on adolescent depression

This study indicates that family adaptability and cohesion have a significant predictive effect on adolescent depression. family adaptability and cohesion have a significant negative correlation with adolescent depression, which means that the higher the family adaptability and cohesion, the lower the depression levels in teenagers. This research result is consistent with the conclusions drawn by previous related studies (41), thereby confirming Hypothesis 1. Family adaptability and cohesion are important predictors of adolescent mental health, with individuals who experience higher levels of family closeness being less likely to exhibit internalizing problems (42). Wang Yao and Wang Yulong conducted a study on 481 adolescents to investigate the relationship between family adaptability and cohesion and their mental health (43). The study found that both family adaptability and cohesion have a significant positive predictive effect on adolescents’ mental health. Meanwhile, the circumplex model theory of family functioning also suggests that family closeness is closely related to the mental health of family members, and low levels of closeness may increase the risk of depression (7). One possible explanation is that members of low-cohesion families often lack sufficient emotional support and positive interaction. When adolescents encounter setbacks, they have no one to confide in, which may lead to feelings of loneliness and helplessness, causing negative emotions to accumulate over time and triggering depressive moods (44). Additionally, low-cohesion families often involve high conflict (such as parental arguments) or emotional coldness. Both of these patterns activate adolescents’ stress systems (such as elevated cortisol levels), thereby increasing susceptibility to depression (45). Conversely, in families with higher cohesion, close relationships and high levels of mutual support provide adolescents with sufficient security, alleviating feelings of loneliness. Appropriate family cohesion also offers adequate personal space, providing emotional support while allowing adolescents to develop autonomously, thereby enhancing psychological resilience and reducing depression levels (46).

### The separate mediating roles of PSS and coping styles

The findings of this study indicate that PSS, positive coping, and negative coping each play a partial mediating role in the relationship between family adaptability and cohesion and adolescent depression, thereby confirming Hypothesis 2 and Hypothesis 3. Family Systems Theory posits that higher levels of family cohesion facilitate the development of healthy social support perception among family members (10). Empirical research further demonstrates that family cohesion significantly and positively predicts adolescents’ social adaptation (47). Adolescents who perceive their families as having lower cohesion and adaptability often struggle to form strong social connections (48), resulting in lower levels of PSS. In contrast, families with high cohesion significantly enhance adolescents’ psychological security by fulfilling their needs for emotional support and belonging (49), thereby increasing their PSS. Concurrently, research suggests that adolescents with higher PSS experience more positive emotions (12). As a critical mediator between family cohesion/adaptability and adolescent depression, social support effectively buffers the negative psychological impact of stressful events by providing emotional care, a sense of belonging, identity affirmation, and instrumental assistance. Consequently, adolescents from families with higher adaptability and cohesion perceive greater social support. This support network provides warmth and encouragement, fosters positive emotions, reduces negative affect, and ultimately leads to lower levels of adolescent depression (50).

Furthermore, the mediating effects of positive coping styles align with previous research findings (51). According to Family Systems Theory (10), the family constitutes a dynamic emotional system in which the level of cohesion influences how members cope with stress. Adolescents in families with high cohesion tend to adopt positive coping strategies, such as problem-solving or seeking support, enabling them to confront challenges proactively. Research has shown that employing positive coping approaches, such as sharing feelings or seeking help, facilitates emotional support and helps alleviate negative emotions in adolescents (52). Thus, positive coping styles are significantly negatively correlated with adolescent depression. Notably, the mediating effect of negative coping styles is somewhat inconsistent with previous research, as this study found a positive correlation between family adaptability and cohesion and negative coping. This may be because adolescents are in a critical period of development, and overly close family relationships may hinder the development of their autonomy (53), making it difficult for them to effectively solve problems when facing difficulties, thus leading them to adopt escape and other negative coping strategies. Such negative coping methods like avoidance or self-blame not only inhibit adolescents’ problem-solving abilities but also increase their psychological stress and exacerbate negative emotions (54), showing a significant positive correlation with depression.

### The chain-mediated role of PSS and coping styles in family adaptability and cohesion in relation to adolescent depression

This study established a chain mediation model to elucidate the relationship between family adaptability and cohesion and adolescent depression, detailing the chain-mediated roles of PSS, positive coping styles, and negative coping styles within this relationship. These findings support Hypothesis 4. According to the buffering model (15), the family serves as the most central source of social support for adolescents, and its level of cohesion is closely associated with adolescents’ subjective perception of social support. In families with high cohesion, adolescents tend to experience stronger social support and are more inclined to use positive coping strategies, such as actively attempting to solve problems. These adolescents also generally exhibit relatively lower levels of depression. This mechanism may be attributed to the family’s role as the primary setting for socialization, where its level of intimacy shapes adolescents’ cognitive schemas regarding social relationships. In highly cohesive families, close emotional communication and supportive behaviors among members help foster positive social beliefs in adolescents, making them more inclined to perceive and accept social support from the external environment (55). When facing stressful situations, adolescents with access to a greater quantity and variety of social support tend to develop stronger confidence in their own coping abilities (56). As a result, they are more likely to choose positive coping strategies to address problems, buffer negative emotions, and maintain lower levels of depression.

Meanwhile, the results of this study also indicate that family adaptability and cohesion, as well as PSS, are positively correlated with negative coping strategies. Negative coping strategies, in turn, are positively associated with adolescent depression. In other words, adolescents with higher family cohesion, who also perceive higher social support, may sometimes be more inclined to adopt negative coping strategies such as avoidance when facing problems, which ultimately increases their risk of depression. This finding diverges from prior literature. A potential reason for this discrepancy is parental overprotection. Research has found that adolescents with high levels of family cohesion and PSS may have experienced a certain degree of parental overprotection during their upbringing (57). In such an environment, although adolescents feel supported by their families, they lack opportunities to independently confront challenges and fail to adequately develop problem-solving skills. Consequently, they tend to rely on avoidant and dependent negative coping strategies to evade problems, which can exacerbate their experience of stress and ultimately elevate their risk of depression (58). Furthermore, under the influence of sociocultural expectations, males are often encouraged to cultivate qualities such as “toughness” and “independence” throughout their socialization. This conditioning fosters an earlier tendency to engage in proactive problem-solving, making them more likely to employ problem-focused positive coping strategies when confronted with stress. By contrast, females are typically afforded greater freedom to express emotions. As a result, when facing difficulties, they may lean more toward emotional expression or avoidant coping styles, rather than taking direct action to address the problem itself (59). Given the gender imbalance in the sample, the observed gender difference could also contribute to variations in the correlation patterns observed between variables.

Overall, this research reveals the psychological mechanisms by which family adaptability and cohesion influence adolescent depression: family adaptability and cohesion affect adolescent depression. This study not only deepens a systematic understanding of the causes of depression in adolescents, but also provides a multi-level (family, social, and individual) intervention blueprint that can help reduce depression levels in adolescents, reduce suicide risks, and protect their physical and mental health.

## Research significance and limitations

### Theoretical significance

The purpose of this study is to explore the relationship between family adaptability and cohesion, PSS, coping styles and adolescent depression, and to deeply analyze the intrinsic mechanism of action between them to provide theoretical reference for the prevention and intervention of adolescent depression. In addition, this study can enrich and improve the existing research system on family adaptability and cohesion, PSS, coping styles, and adolescent depression. It explores in depth the intrinsic mechanism of family adaptability and cohesion affecting adolescent depression, which is an important supplement to previous theoretical research (31–33). This research lays a solid theoretical foundation for the development of scientific, effective and targeted prevention and intervention measures, and is significant for alleviating depressive symptoms among adolescents and maintaining their mental health.

### Practical significance

In practice, mental health issues among adolescents are becoming increasingly prominent, with depression emerging as one of the primary obstacles to their healthy development (60). This study, by constructing a chain mediation model of “family adaptability and cohesion → PSS → coping styles → adolescent depression,” integrates two mediating pathways and organically synthesizes factors at the family, social, and individual levels to systematically examine their combined effects and interactions. This framework helps to reduce the likelihood of adolescent depression, enables early identification, and supports the formulation of effective intervention measures to mitigate the risks of long-term depression and suicide, thereby safeguarding adolescent mental health (61, 62).

### Limitation

Although this study examined the relationships among these four variables, research on intervention measures and effective methods targeting these factors remains relatively limited. Future efforts should focus on how intervention measures can enhance family adaptability and cohesion, strengthen social support, and improve coping styles to reduce the incidence of depression. For example, interventions such as group counseling and mindfulness training can be implemented. These aim to reduce adolescents’ negative stress responses, enhance their ability to choose positive coping strategies, and thereby effectively lower their depression levels. Second, the measurement of all variables is entirely based on self-reported questionnaire surveys, and participants may have withheld information. Furthermore, in this study, gender and primary caregiver gender were included as control variables in the mediation analysis, which did not allow for an in-depth exploration of potential gender differences in the mediation pathways. Future research could further examine whether the individual pathways are gender-specific. Meanwhile, the gender ratio in the study sample was imbalanced, which may have affected the stability and representativeness of the overall analysis to some extent. Future research could further examine the relationships among the variables in more gender-balanced samples to enhance the generalizability of the findings. Finally, although this study was based on solid theoretical and empirical foundations, the cross-sectional nature restricted my capacity to ascertain causal links among the variables. Future research can use longitudinal tracking methods to more clearly reveal the correlation between these variables.

## Conclusion

The results of this study show that

1. Family adaptability and cohesion can negatively predict depression in adolescents. The closer the family relationship and the stronger the adaptability of individuals, the lower the degree of depression.
2. Family adaptability and cohesion, PSS, positive coping styles, and negative coping styles are correlated with adolescent depression.
3. Family adaptability and cohesion have an indirect impact on adolescent depression through PSS and the chain-mediated role of coping styles. That is to say, teenagers with higher levels of family adaptability and cohesion usually feel more social support and are more inclined to adopt positive coping styles, thereby reducing the risk of developing depression. Meanwhile, adolescents with stronger family cohesion and greater social support may also be more prone to adopting negative coping strategies, which ultimately heightens their risk of depression.

## Data Availability

The original contributions presented in the study are included in the article/supplementary material. Further inquiries can be directed to the corresponding author/s.

## References

[1] Paykel ES Scott J Cornwall PL Abbott R Crane C Pope M . Duration of relapse prevention after cognitive therapy in residual depression: follow-up of controlled trial. Psychol Med. (2005) 35:59–68. doi: 10.1017/S003329170400282X, PMID: 15842029

[2] Deng YG Shen LL . Impact of social network usage on depression in adolescents. Chin J Sch Health. (2023) 44:627–31. doi: 10.16835/j.cnki.1000-9817.2023.04.034

[3] Tong Y Xie F Wen X Li Y Yuan M Zhang X . Longitudinal association between bullying victimization and depressive symptoms in Chinese early adolescents: the effect of life satisfaction. Depress Anxiety. (2024) 2024:6671415. doi: 10.1155/2024/6671415, PMID: 40226671 PMC11918514

[4] Thapar A Collishaw S Pine DS Thapar AK . Depression in adolescence. Lancet. (2012) 379:1056–67. doi: 10.1016/S0140-6736(11)60871-4, PMID: 22305766 PMC3488279

[5] Hirschfeld RM Montgomery SA Keller MB Kasper S Schatzberg AF Möller HJ . Social functioning in depression: a review. J Clin Psychiatry. (2000) 61:268. doi: 10.4088/JCP.v61n0405, PMID: 10830147

[6] Leung C Lo SK Tsang S Chan R Kung E . The relationship between family dining practices, parenting style and family functioning and child learning. Int J Disabil Hum Dev. (2016) 15:267–76. doi: 10.1515/ijdhd-2015-0013

[7] Olson DH . Circumplex model VII: validation studies and FACES III. Fam Process. (2010) 25:337–51. doi: 10.1111/j.1545-5300.1986.00337.x, PMID: 3758310

[8] Anyan F Hjemdal O . Stress of home life and gender role socializations, family cohesion, and symptoms of anxiety and depression. Women Health. (2018) 58:548–64. doi: 10.1080/03630242.2017.1316343, PMID: 28379114

[9] Dahlem NW Zimet GD Walker RR . The Multidimensional Scale of Perceived Social Support: a confirmation study. J Clin Psychol. (1991) 52:756–61. doi: 10.1002/1097-4679(199111)47:63.0.CO;2-L 1757578

[10] Bowen M . Family therapy in clinical practice. Family Process. (2010) 19:87–96. doi: 10.1111/j.1545-5300.1980.087_1.x

[11] He XZ You HY . Correlation of family cohesion, family adaptability and perceived social support. Chin J Rehabil Theory Pract. (2006) 12:634–6. Available online at: https://center.ras.metaersp.cn:17105/s/net/cnki/kns/G.https/nzkhtml/xmlRead/trialRead.html?dbCode=CJFD&tableName=cjfd2006&fileName=ZKLS200607036&fileSourceType=1&appId=KNS_BASIC_PSMC&invoice=KMVFcFWpUREX3saxRtDGuOcdyWwaZd17uDpbd+W/w5D9VRnZ0Ieblbz1ql8SdOpkCFqy+qDVXduxE/Gf8prFK7YrNpCCBRHMKkuSKqTJ8adVYXYisDTvTkWMwL3czicsXEQu24WOg2CKMmr/rOhjpcpxpjwgbfBBTdEVd2OFy7w=&platform=NZKPT&type=JOURNAL&cflag=html&trial=&nonce=05610729B98144C69B9AA91A78897451&;x-chain-id=b6hemnr11lhc

[12] Liu H Wen S . Family cohesion and depression on adolescents: A mediating model of perceived social support and self-esteem. Adv Educ Technol Psychol. (2024) 8:65–72. doi: 10.23977/aetp.2024.080108

[13] Wang J Guan X Zhang Y Li Y Ahmed MZ Jobe MC . Effect of family cohesion on depression of chinese college students in the COVID-19 pandemic: chain mediation effect of perceived social support and intentional self-regulation. Int J Ment Health Promot. (2023) 25:223–35. doi: 10.32604/ijmhp.2022.025570

[14] Barragán Martín AB Molero Jurado MDM Pérez-Fuentes MDC Oropesa Ruiz NF Martos Martínez Á Simón Márquez MDM . Interpersonal support, emotional intelligence and family function in adolescence. Int J Environ Res Public Health. (2021) 18:5145. doi: 10.3390/IJERPH18105145, PMID: 34066285 PMC8152060

[15] Cohen S Wills TA . Stress, social support, and the buffering hypothesis. Psychol Bull. (1985) 98:310–57. doi: 10.1037/0033-2909.98.2.310 3901065

[16] Jin TL Wu YTN Zhang L Yang X Jia YR Yang H . The effect of perceived chronic social adversity on aggression of college students: the roles of ruminative responses and perceived social support. Psychol Dev Educ. (2020) 36:8. doi: 10.16187/j.cnki.issn1001-4918.2020.04.04

[17] Ye BJ Ma TT . The relationship between social support and college students’ Depression: A moderated mediation model. Psychol Explor. (2020) 40:465–71. Available online at: https://center.ras.metaersp.cn:17105/s/net/cnki/kns/G.https/nzkhtml/xmlRead/trialRead.html?dbCode=CJFD&tableName=cjfdlast2020&fileName=XLXT202005013&fileSourceType=1&appId=KNS_BASIC_PSMC&invoice=QUCYOxBSTrNWtZhH7pZtlrt0n9VUoi0Lt+Y3PgNSi/XF52DlyIb/xCtr7470xVunDK0BnqF6ou36zhn/yQF3FbBHHAzWPeVJCO6nRHTsI32NpLv/VMPBGuNFCxH+gUeG0fWs3KxHw/g7m7DwBRzZVqsoVHq/eixwwAmBm4ugF7Y=&platform=NZKPT&type=JOURNAL&cflag=html&trial=&nonce=02BCB0189FD740B3BB389F7155DA3692&;x-chain-id=b6hfkyzuevb4

[18] Branje S Hale WW Frijns T Meeus WHJ . Longitudinal associations between perceived parent-child relationship quality and depressive symptoms in adolescence. J Abnorm Child Psychol. (2010) 38:751–63. doi: 10.1007/s10802-010-9401-6, PMID: 20217211 PMC2902740

[19] Wang LY . The Effect of growth mindset and intervention on procrastination behavior: the chain mediating role of self-efficacy and coping style. Lanzhou, Gansu, China: Northwest Normal University. [Master’s thesis]. (2024). doi: 10.27410/d.cnki.gxbfu.2024.002355

[20] Lazarus R Folkman S . Stress, appraisal and the coping process (1984). Available online at: https://www.scienceopen.com/document?vid=633969a3-1339-4698-8081-925591cc1b35 (Accessed February 12, 2026).

[21] Compas BE Champion JE Forehand R Cole DA Reeslund KL Fear J . Coping and parenting: Mediators of 12-month outcomes of a family group cognitive–behavioral preventive intervention for families of depressed parents. J Consult Clin Psychol. (2010) 78:623–34. doi: 10.1037/a0020459, PMID: 20873898 PMC3030923

[22] Altiere MJ Kluge SV . Family functioning and coping behaviors in parents of children with autism. J Child Fam Stud. (2009) 18:83. doi: 10.1007/s10826-008-9209-y

[23] Beck AT . Cognitive therapy and the emotional disorder. New York: Int Universities Press. (1976) 356. Available online at: https://psycnet.apa.org/record/1976-28303-000

[24] Wang WL . The impact of psychological capital on psychological symptoms among college students: the multiple mediating effects of coping style. J High Educ. (2022) 8:81–184+188. doi: 10.19980/j.CN23-1593/G4.2022.17.045

[25] Faulk KE Gloria CT Steinhardt MA . Coping profiles characterize individual flourishing, languishing, and depression. Anxiety Stress Coping. (2013) 26:378–90. doi: 10.1080/10615806.2012.708736, PMID: 22853921

[26] Zhang YJ Yan KL Wang JL . A path analysis on life events, negative automatic thoughts, coping style and depression. Psychol Dev Educ. (2005) 21:96–9. doi: 10.3969/j.issn.1001-4918.2005.01.018

[27] Zhang Z . Mediation of perceived social support in personality traits and positive coping style among post-2000 college students. Chin J Health Psychol. (2020) 28:1238–42. doi: 10.13342/j.cnki.cjhp.2020.08.027

[28] Lu WF Huang XY Li WC Wu LL Gan Y . Comprehending the impact of social support on post-stress growth among higher vocational students: The parallel mediating roles of positive and negative coping. Psychol. (2024) 19:81–4. doi: 10.19738/j.cnki.psy.2024.08.022

[29] Sarason IG Sarason BR Pierce GR . Social support: the search for theory. J Soc Clin Psychol. (1990) 9:133–47. doi: 10.1521/jscp.1990.9.1.133

[30] Tang JY Fu CY Xu W . The influence of family function on adolescents’ Depression and social anxiety: the mediating role of belief in a just world. Psychol Dev Educ. (2025) 41:701–9. doi: 10.16187/j.cnki.issn1001-4918.2025.05.10

[31] Yap MBH Pilkington PD Ryan SM Jorm AF . Parental factors associated with depression and anxiety in young people: a systematic review and meta-analysis. J Affect Disord. (2014) 156:8–23. doi: 10.1016/j.jad.2013.11.007, PMID: 24308895

[32] Hou JQ Chen ZY . The trajectories of adolescent depressive symptoms: Identifying latent subgroups and risk factors. Acta Psychol Sin. (2016) 48:957–68. doi: 10.3724/SP.J.1041.2016.00957

[33] Rueger SY Malecki CK Pyun Y Aycock C Coyle S . A meta-analytic review of the association between perceived social support and depression in childhood and adolescence. Psychol Bull. (2016) 142:1017–67. doi: 10.1037/bul0000058, PMID: 27504934

[34] Platt J Prins S Bates L Keyes K . Unequal depression for equal work? how the wage gap explains gendered disparities in mood disorders. Soc Sci Med. (2016) 149:1–8. doi: 10.1016/j.socscimed.2015.11.056, PMID: 26689629 PMC4801117

[35] Davis-Kean and Pamela E . The influence of parent education and family income on child achievement: the indirect role of parental expectations and the home environment. J Fam Psychol. (2005) 19:294–304. doi: 10.1037/0893-3200.19.2.294, PMID: 15982107

[36] Cochran WG . Sampling Techniques. 3rd ed. New York, NY: John Wiley & Sons (1977).

[37] Fei LP Shen QJ Zheng YP Zhao JP Jiang SA Wang LW . Preliminary evaluation of Chinese version of FACES II and FES: comparision of normal families and families of schizophrenic patients. Chin Ment Health J. (1991) 5:198–202. Available online at: https://center.ras.metaersp.cn:17105/s/net/cnki/kns/G.https/reader/flowpdf?invoice=gQk0D7MjVV6932BGZKCe7Y%2B8KhDYV5l6NIbfxo8prYgyqvO43K0XG1vdvG7pMHT%2FG8ZDSxoSJ8AuI%2BW2cTLXoS99xwsZGbE6iaGVAVSlVAQC42QhPc3H4WhiwgHQpWMPEN%2FZ3BzXJvVLHysVXW7xi5GdDCl4GcYtn4z0IowZMk4%3D&platform=NZKPT&sourcetype=nxgp&product=CJFQ&filename=ZXWS199105001&tablename=cjfd9093&type=JOURNAL&scope=trial&cflag=overlay&dflag=pdf&pages=&language=CHS&trial=&nonce=D454DF54E05649CBA2F68DCDAD39FA34

[38] Zimet GD Dahlem NW Zimet SG Farley GK . The multidimensional scale of perceived social support. J Pers Assess. (1988) 52:30–41. doi: 10.1207/s15327752jpa5201_2, PMID: 2280326

[39] Xie YN . A preliminary study on the reliability and validity of the Simple Coping Style Scale. Chin J Clin Psychol. (1998) 6:114–5. doi: 10.16128/j.cnki.1005-3611.1998.02.018

[40] Arrieta J Aguerrebere M Raviola G Flores H Elliott P Espinosa A . Validity and utility of the patient health questionnaire (PHQ)-2 and PHQ-9 for screening and diagnosis of depression in rural chiapas, Mexico: A cross-sectional study. J Clin Psychol. (2017) 73:1076–90. doi: 10.1002/jclp.22390, PMID: 28195649 PMC5573982

[41] Yang DH . Relationship between depression and anxiety of middle school students and the degree of intimacy and adaptability in the family. Health Psychol J. (2001) 9:417–9. doi: 10.3969/j.issn.1005-1252.2001.06.009

[42] Deng S Lopez V Roosa WM Ryu E Burrell GL Tein JY . Family processes mediating the relationship of neighborhood disadvantage to early adolescent internalizing problems. J Early Adolesc. (2006) 26:206–31. doi: 10.1177/0272431605285720

[43] Wang Y Wang YL . Mediating role of father-child attachment between family functioning and adolescents’ emotional health and its gender differences. Chin J Health Psychol. (2020) 28:150–5. doi: 10.13342/j.cnki.cjhp.2020.10.033

[44] Wang D Zhu KM Li P Lv F Yang Y . A study of relationship between the loneliness and depressive emotion of college students. Med J Chin People Health. (2003) 15:18–20. doi: 10.3969/j.issn.1672-0369.2003.01.008

[45] Repetti RL Taylor SE Seeman TE . Risky families: family social environments and the mental and physical health of offspring. Psychol Bull. (2002) 128:330–66. doi: 10.1037//0033-2909.128.2.230, PMID: 11931522

[46] Xiong YM Li F Sheng XC . A study of relationship between the loneliness and depressive emotion of college students. Chin J Health Psychol. (2013) 21:449–51. doi: 10.13342/j.cnki.cjhp.2013.03.063

[47] Kurock R Gruchel N Bonanati S Buhl HM . Family climate and social adaptation of adolescents in community samples: A systematic review. Adolesc Res Rev. (2022) 7:551–63. doi: 10.1007/s40894-022-00189-2

[48] Wentzel KR Feldman SS . Relations of cohesion and power in family dyads to social and emotional adjustment during early adolescence. J Res Adolesc. (1996) 6:225–44. doi: 10.1111/j.1752-0606.1996.tb00195.x

[49] Baumeister RF Leary MR . The need to belong: desire for interpersonal attachments as a fundamental human motivation. Psychol Bull. (1995) 117:497–529. doi: 10.1037/0033-2909.117.3.497 7777651

[50] Tian M Wang JJ Song GW . Harsh parenting and adolescents’ Depression: A moderated mediation model. Chin J Spec Educ. (2018) 25:71–7. doi: 10.3969/j.issn.1007-3728.2018.06.013

[51] Zheng RZ Dong YH Li J Dong Q Liu JJ Huang F . Correlation between depression and familial environment and coping style among middle school students. Chin J Public Health. (2012) 28:1280–2. doi: 10.11847/zgggws2012-28-10-06

[52] Compas BE Connor-Smith JK Saltzman H Thomsen AH Wadsworth ME . Coping with stress during childhood and adolescence: problems, progress, and potential in theory and research. Psychol Bull. (2001) 127:87–127. doi: 10.1037/0033-2909.127.1.87, PMID: 11271757

[53] Buehler BC . Family cohesion and enmeshment: different constructs, different effects. J Marriage Fam. (1996) 58:433–41. doi: 10.2307/353507

[54] Nolen-Hoeksema S . Responses to depression and their effects on the duration of depressive episodes. J Abnorm Psychol. (1991) 100:569. doi: 10.1037//0021-843X.100.4.569, PMID: 1757671

[55] Hedges D . Attachment and loss.,” Poetry, therapy and emotional life. CRC Press. (2017) . p:51–62. doi: 10.1201/9780203743041-5

[56] Bonino S . Self-efficacy. The exercise of control. In: Coping with chronic illness. London: Routledge (2020). p. 33–7. doi: 10.4324/9780367822231-10

[57] Padilla-Walker LM Nelson LJ . Black hawk down?: establishing helicopter parenting as a distinct construct from other forms of parental control during emerging adulthood. J Adolesc. (2012) 35:1177–90. doi: 10.1016/j.adolescence.2012.03.007, PMID: 22503075

[58] Lin J Tu W . Relationship among positive psychological qualities, coping styles and perceived social support in college students. Chin J Health Psychol. (2015) 23:225–8. doi: 10.13342/j.cnki.cjhp.2015.02.020

[59] Tamres LK Janicki D and Helgeson VS . Sex differences in coping behavior: A meta-analytic review and an examination of relative coping. Pers Soc Psychol Rev. (2002) 6:2–30. doi: 10.1207/s15327957pspr0601_1

[60] Shi LJ Li YT Li YT Cao LY Gong JB . Longitudinal relationships between depression, social anxiety, and internet addiction in adolescents: A cross-lagged panel network analysis. Chin J Clin Psychol. (2025) 33:1034–41. doi: 10.16128/j.cnki.1005-3611.2025.05.024

[61] Wang LQ Li Q Gu C Zhu HQ . Relationships between depression with family intimacy and adaptability among adolescents. Chin J Sch Health. (2017) 38:1650–2. doi: 10.16835/j.cnki.1000-9817.2017.11.015

[62] Wu YN Zhao GP . Perceived social support and depression among vocational students: a chain mediating effect of meaning in life and coping styles. Psychol Mag. (2024) 19:58–62. doi: 10.19738/j.cnki.psy.2024.11.014

